# Endometrial Endometrioid Carcinoma Metastases Show Decreased ER-Alpha and PR-A Expression Compared to Matched Primary Tumors

**DOI:** 10.1371/journal.pone.0134969

**Published:** 2015-08-07

**Authors:** Carla Bartosch, Sara Monteiro-Reis, Renata Vieira, Armindo Pereira, Marta Rodrigues, Carmen Jerónimo, José M. Lopes

**Affiliations:** 1 Department of Pathology, Portuguese Oncology Institute-Porto, Porto, Portugal; 2 Cancer Biology and Epigenetics Group, Research Center of Portuguese Oncology Institute- Porto, Porto, Portugal; 3 Medical Faculty, University of Porto, Porto, Portugal; 4 Department of Pathology, Centro Hospitalar São Joao, Porto, Portugal; 5 Department of Pathology and Molecular Immunology, Institute of Biomedical Sciences Abel Salazar-ICBAS, University of Porto, Porto, Portugal; 6 IPATIMUP, Institute of Molecular Pathology and Immunology, Porto, Portugal; Michigan State University, UNITED STATES

## Abstract

Patients with endometrial endometrioid carcinoma (EEC) that present with advanced primary disease and develop recurrences have a poor outcome. The phenotype of EEC metastases and recurrences is poorly studied. We evaluated the morphological features and ER-alpha/PRA/p53 immunohistochemical expression of a sample of 45 EEC metastases compared to matched primary tumors. Additionally, we studied methylation levels of ER-alpha/PRA gene promoters. The distribution of histological FIGO grade was significantly different in metastases, which disclosed higher grade than primary tumors (p = 0.005). Mitotic index was significantly lower in metastases compared to matched primary tumors (p<0.001). ER-alpha (p = 0.002) and PRA (p<0.001) median H-scores were significantly lower in metastases than in matched primary EECs, but there was no significant difference concerning p53 expression (p = 0.056). ER-alpha/PRA expression differences did not correlate with differences in metastases morphology. ER-alpha/PRA gene promoter levels were globally low (range: 0% to 11.9%). One case showed higher ER-alpha gene promoter methylation in metastasis compared to matched EEC primary tumor. Regarding PRA, there was a significant higher frequency of its promotor methylation in metastases compared to primary tumors (51.6% *vs*. 22.7%, p = 0.022). In conclusion, EEC metastatic disease displays phenotypic changes along with ER-alpha and PRA decreased expression compared to primary tumors. ER-alpha and PRA gene promoter methylation seems to play a limited role in the etiology of these alterations. PR expression assessment for hormonal treatment decision of patients with advanced tumors, may be more adequate in metastases than in EEC primary tumors.

## Introduction

In general, patients with endometrial endometrioid carcinoma (EEC) have a good prognosis. However, approximately 25% of patients present with advanced primary disease outside the uterus at the time of diagnosis, and 15–20% of patients have recurrences later in the course of disease [1, 2]. Most patients with advanced and recurrent EEC will die of disease, and death rates are reported to be increasing [3, 4]. Characterization of EEC metastases and recurrences is poorly studied.

Steroid hormones, namely estrogens and progestins, play a major role in EEC. Estrogens have been associated with endometrial cancer development and growth, while progestins have an opposite effect, promoting differentiation and growth inhibition [5]. The effects of both hormones in EEC are mediated by their nuclear receptors. Loss of estrogen receptor (ER) and progesterone receptor (PR) expression, as well as p53 expression, have been associated with aggressive phenotypes and poor survival in EEC primary tumors [2, 6, 7]. Gene promotor hypermethylation has been suggested as one of the mechanisms responsible for ER and PR expression loss [8–12]. Furthermore, the expression levels of each of the ER subtypes (alpha and beta) and the different PR isoforms (A and B), seem to regulate proliferation and differentiation of endometrial cells, and were postulated to be altered in endometrial cancer [13–16].

Patients with advanced or recurrent EEC might be candidates for hormone therapy, being the response to hormone treatment dependent on PR expression [5]. However, assessment of hormone receptor expression is usually not routinely performed before treatment initiation, and when requested is usually performed in the primary tumor. Although studies on ER and PR expression in EEC metastases or recurrences are limited, it has been suggested that hormone receptor expression diminishes in metastatic disease [17–19].

In this study we aimed to evaluate the morphological phenotype and ER/PR/p53 immunohistochemical expression of a sample of EEC metastases compared to matched primary tumors. Additionally, we studied the methylation levels of ER and PR gene promoters both in EEC primary tumors and corresponding metastases.

## Methods

### Patients and histological evaluation

We identified EEC patients diagnosed with metastasis at two Portuguese tertiary centers [Centro Hospitalar S. João (CHSJ) and Portuguese Institute of Oncology—Porto (IPOP)], between 2000 and 2013, by searching the respective Pathology Department databases.

Cases with histological confirmation of metastasis, either at diagnosis or during follow-up, were selected. Cases with ambiguous histology and synchronous ovarian endometrioid carcinoma, only local vaginal recurrence, cytological confirmation of metastasis or unavailable formalin fixed paraffin embedded (FFPE) tissue, were excluded. Altogether, 45 pairs of EEC primary tumors and metastases were included in the study, after approval by the Ethics Committee of both participating institutions [CHSJ (CES44/2010)/IPOP (CES494-010)]. Because this was a retrospective study, based on archive paraffin-embedded tissue and many patients were deceased when the study was started, no informed consent was procured and data was anonymized and de-identified prior to analysis.

The patients’ clinical files and pathological material were retrospectively reviewed. Data collected included patients’ age at diagnosis, tumor size, histological subtype, lymphovascular invasion, International Federation of Gynecology and Obstetrics (FIGO) stage, treatment (radiotherapy, hormone therapy and chemotherapy), site of metastases, progression free and overall survival.

All histological slides were reviewed by a gynecopathologist (CB). Histological subtype, histological FIGO grade (according to the WHO criteria) and mitotic index [number of mitosis per 10 high power fields (HPF)] were evaluated both in primary tumors and metastases.

### Immunohistochemistry

ER, PR and p53 protein expression was assessed by immunohistochemistry. Selected FFPE blocks were sectioned at 3 μm thickness. Sections were deparaffinized in xylene and rehydrated through a graded ethanol series. Antigen retrieval was achieved by incubating the sections in preheated EDTA buffer (pH 8) for ER-alpha and in citrate buffer (pH6) for PRA and p53 in a water bath (98°C) for 20 minutes. Endogenous peroxidase was blocked and the slides were incubated for 1h using the following antibodies: ER alpha (clone 6F11, mouse monoclonal antibody, 1:100, Novocastra Leica Biosystems), PRA (clone 16, mouse monoclonal antibody, 1:150, Novocastra Leica Biosystems) and p53 (clone DO7, mouse monoclonal antibody, 1:200, Dako). Finally, sections were incubated with Poly-HRP-GAM/R/R IgG (ImmunoLogic) and counterstained with hematoxylin. Appropriate positive and negative controls were also included in each assay.

ER-alpha, PRA and p53 expression was evaluated by optical microscopy using an image analysis program (GenASIs Go-Path). Fifteen random fields of the tumor in each slide (corresponding to a mean of 7138 tumors cells per case) were photographed. In each photo tumor cells were selected and evaluated using H-score. H-score was defined by the intensity grade of staining (0–3) multiplied by the percentage of positive cells, resulting in a range of possible scores of 0–300.

### DNA extraction

Representative sections of EEC primary tumors and matched metastases larger than 5mm (n = 31), were selected for DNA extraction. Enriched tumor cell areas were marked on a HE-stained slide and used as a template for macroscopic dissection. The corresponding areas of ten consecutive 7μm thick sections placed on glass slides were scratched into 1.5mL tubes using a sterile, single-use scalpel. Tissue samples were deparaffinized and then submitted to overnight digestion, in a dried bath incubator at 55°C, using 1mL of digestion buffer (composed by Tris-HCl 1M, EDTA 0,1M, Tween 20 and sterile bidistilled water), and proteinase K (20mg/ml, 50μL) (Sigma-Aldrich, Germany). Genomic DNA was extracted by standard phenol–chlorophorm method, precipitated with ethanol and eluted in 20 μl of water [20].

### Bisulfite modification and quantitative methylation-specific PCR

DNA samples from were subjected to sodium bisulfite modification, using the EZ DNA Methylation-Gold Kit (Zymo Research, Orange, CA, USA) according to manufacturer’s instructions.

Methylation analysis of estrogen (ESR1) and progesterone (PRA) receptor gene promoters was performed by quantitative methylation-specific PCR (qMSP). Bisulfite-modified genomic DNA was tested using four sets of primers that recognize the methylated sequence located at the promoter regions of ERS1, and PRA genes. Primers used were those previously published [10, 21]: PRA forward: 5’-ACGGGTTATTTTTTTTTCG-3’, reverse: 5’-TAAAATATACGCCCTCCACG-3’; ESR1 forward 5’-GGCGTTCGTTTTGGGATTG-3’, reverse 5’-GCCGACACGCGAACTCTAA-3’.

The qMSP was performed in a 10μl reaction volume including 10 μM forward and reverse primers, 50 ng bisulfite treated DNA from tissue samples, and 5μL Kapa Sybr Fast qPCR master mix (Kapa Byosystems). Each sample was analyzed in triplicate in 384-well plates using the LightCycler 480 System (Roche). The geometric mean of the two closest values for each sample was used for data analysis. A standard curve was generated from 1:5 serial dilutions of bisulfite-converted commercially available methylated DNA (CpGenome Universal Methylated DNA; Millipore Billerica). The universal methylated DNA sample was also used as a positive control for the qMSP reaction. Additionally, all plates contained two water blanks as negative controls. All samples were also tested using primers for non–CpG-containing regions of an internal reference gene, β-actin (ACTB) to normalize for input DNA (primers: forward 5’- TGGTGATGGAGGAGGTTTAGTAAGT- 3’, reverse 5’- AACCAATAAAACCTACTCCTCCCTTAA- 3’). The level of methylated DNA (percent of methylated reference, PMR) was calculated using the following formula: [(target gene/ACTB)^sample^ / (target gene/ACTB)^positive control^] x 100. Additionally PMR values were dichotomized to methylated or unmethylated using a cut-off of 4%, as used in a number of prior publications [22, 23].

### Statistical analysis

Data was tabulated and analyzed using STATA (STATACorp, Texas, USA). We performed a pairwise analysis to compare differences between primary tumors and metastases. Marginal homogeneity test was used to compare the distribution of histological FIGO grade across primary tumors and matched metastases. Wilcoxon sign rank test was used to compare median differences in mitotic index, ER-alpha, PRA and p53 expression, and continuous methylation levels between matched primary tumors and metastases. McNemar test was used to compare dichotomized methylation levels between primary tumors and metastases. We used Mann-Whitney and Kruskal-Wallis tests to evaluate the associations of immunohistochemical results with clinical-pathological parameters. Spearman’s rank correlation coefficient was used to evaluate the correlation between ER, PR and mitotic index changes. A p value equal or inferior to 0.05 was considered significant.

## Results

### Clinical and pathological features

The clinicopathological features of our cohort of patients are depicted in Table 1. The mean age of patients was 63.8 years (range: 39–87). All 45 patients included in the study underwent total hysterectomy and the majority (84.5%) was staged as FIGO III/IV. Most (66.7%) underwent adjuvant radiotherapy and 31.1% had adjuvant chemotherapy.

**Table 1:** Clinicopathological features of EEC patients diagnosed with metastases.

**Age (years); mean ± SD**	62.7±9.8
**Hysterectomy; n (%)**	
- Yes	45 (100%)
**Lymphadenectomy; n (%)**	
- Yes	33 (73.3%)
- No	12 (26.7%)
**Omentectomy; n (%)**	
- Yes	8 (17.8%)
- No	37 (82.2%)
**Peritoneal biopsies; n (%)**	
- Yes	10 (22.2%)
- No	35 (77.8%)
**Histological subtype; n (%)**	
- Endometrioid, no specific type	31 (68.9%)
- Endometrioid with squamous differentiation	14 (31.1%)
**Histological FIGO grade; n (%)**	
- Grade 1	16 (35.6%)
- Grade 2	12 (26.7%)
- Grade 3	17 (37.8%)
**Tumor size (cm); mean ± SD**	6.1±2.3
**Lymphovascular invasion; n (%)**	
- Yes	35 (77.8%)
- No	10 (22.2%)
**FIGO stage; n (%)**	
- I	4 (8.9%)
- II	3 (6.7%)
- III	26 (57.8%)
- IV	12 (26.7%)
**Adjuvant radiotherapy; n (%)**	
- Yes	30 (66.7%)
- No	15 (33.3%)
**Adjuvant chemotherapy; n (%)**	
- Yes	14 (31.1%)
- No	31 (68.9%)
**Hormone therapy; n (%)**	
- Yes	12 (26.7%)
- No	22 (48.9%)
- Not available	11 (24.4%)
**Metastasis body site; n (%)**	
- Pelvic lymph nodes	21 (46.7%)
- Para-aortic lymph nodes	6 (13.3%)
- Peritoneum	12 (26.7%)
- Lung	2 (4.4%)
- Supraclavicular/cervical lymph node	2 (4.4%)
- Inguinal lymph node	1 (2.2%)
- Bone	1 (2.2%)

SD—Standard deviation.

Cases were classified as endometrioid carcinomas with no specification (n = 31, 68.9%) and as endometrioid carcinomas with squamous differentiation (n = 14, 31.1%). Focal mucinous differentiation was identified in 5 (11.1%), and 4 (8.9%) had areas of microcystic elongated and fragmented (MELF) pattern. Median tumor size was 6.5 cm (range: 1.4, 11). Thirty (66.7%) invaded more than half of the myometrium, 12 (26.7%) had endocervical stromal invasion and 37 (82.2%) had lymphovascular invasion.

Metastases were diagnosed synchronous to the endometrial carcinoma (n = 28, 62.2%) or later in the course of disease (n = 17, 37.8%). They were located in pelvic/para-aortic lymph (60%) nodes, peritoneum (26.7%), and other extra-pelvic distant sites (13.3%). Nine (20.0%) patients also had local vaginal recurrence. Treatment of metastatic and local recurrent disease included radiotherapy (n = 7, 15.6%), chemotherapy (n = 10, 22.2%) and hormone therapy (n = 12, 26.7%).

At the end of the study 20 (44.4%) patients had died of disease, 1 (2.22%) died of other causes, 23 (51.1%) were alive, and 1 (2.22%) was lost to follow-up. The median follow-up time for survivors was 33.9 months, ranging from 7 months to 11 years. The median disease free survival was 50.9 months (95% CI 29.0–69.2). The overall median survival was 56.7 months (95% CI 40.1–133.5) with a 5-year survival rate of 49.3%.

### Histological comparison of primary tumors and metastases

**Fig 1:**
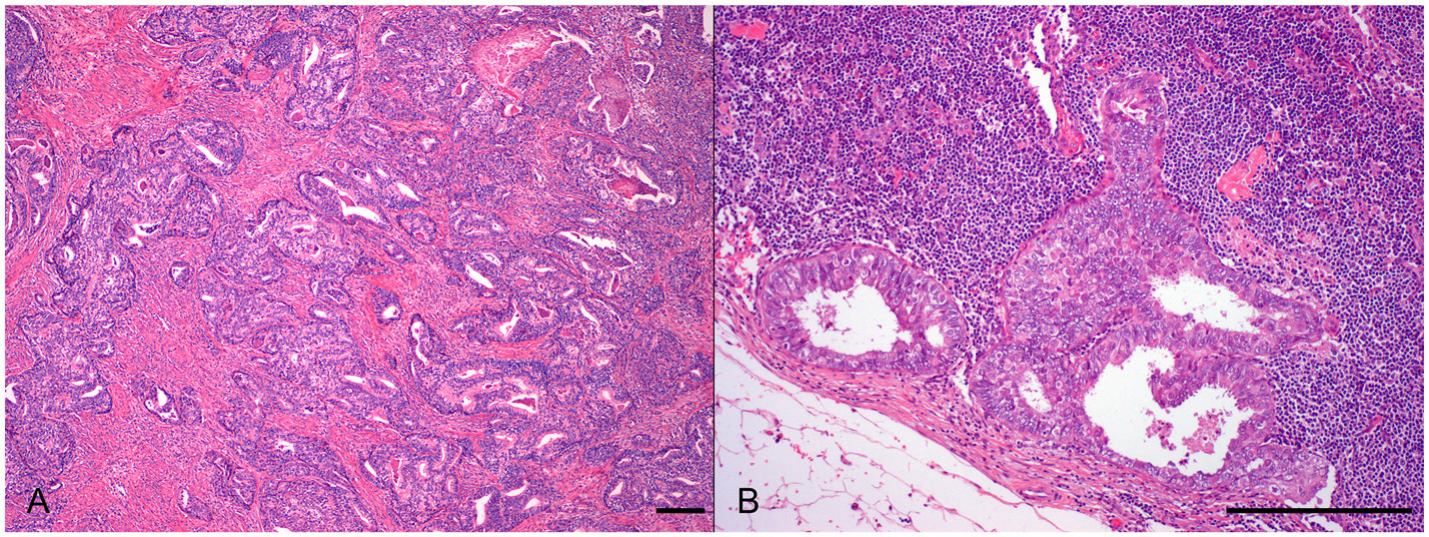
Histological features. Morphological similar EEC primary tumor (A) and matched lymph node metastasis (B). Scale bar = 200μm.

**Fig 2:**
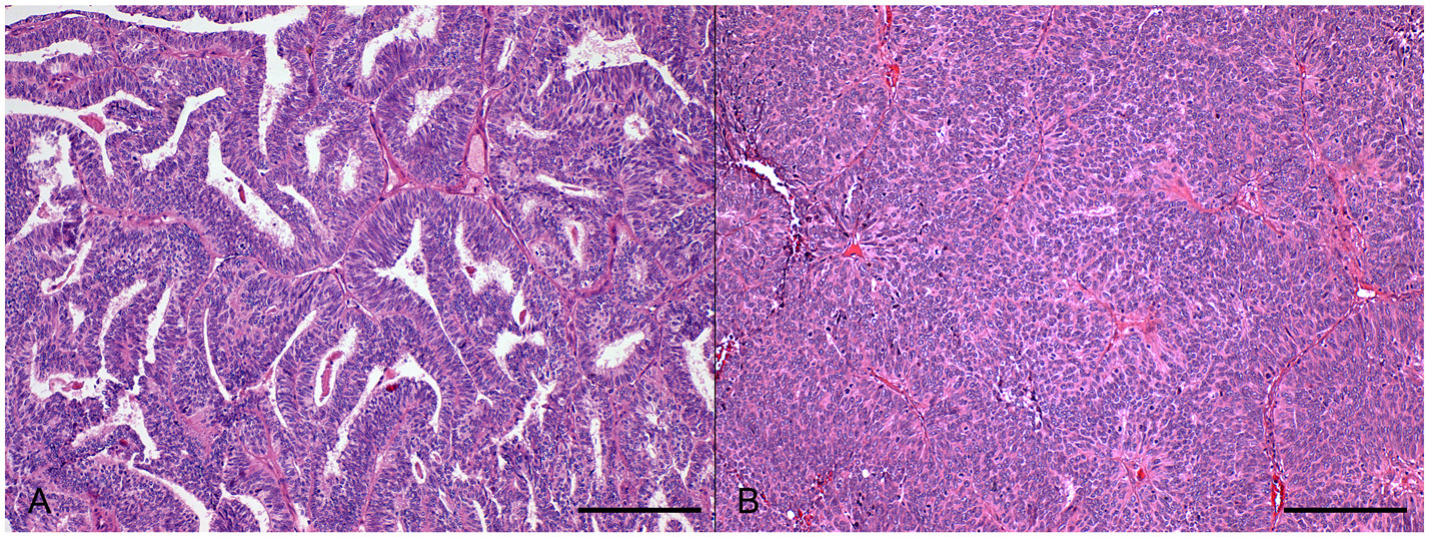
Histological features. Morphological discordant EEC primary tumor (A) and matched peritoneal recurrence (B), showing higher histological FIGO grade in the recurrence. Scale bar = 100μm.

**Fig 3:**
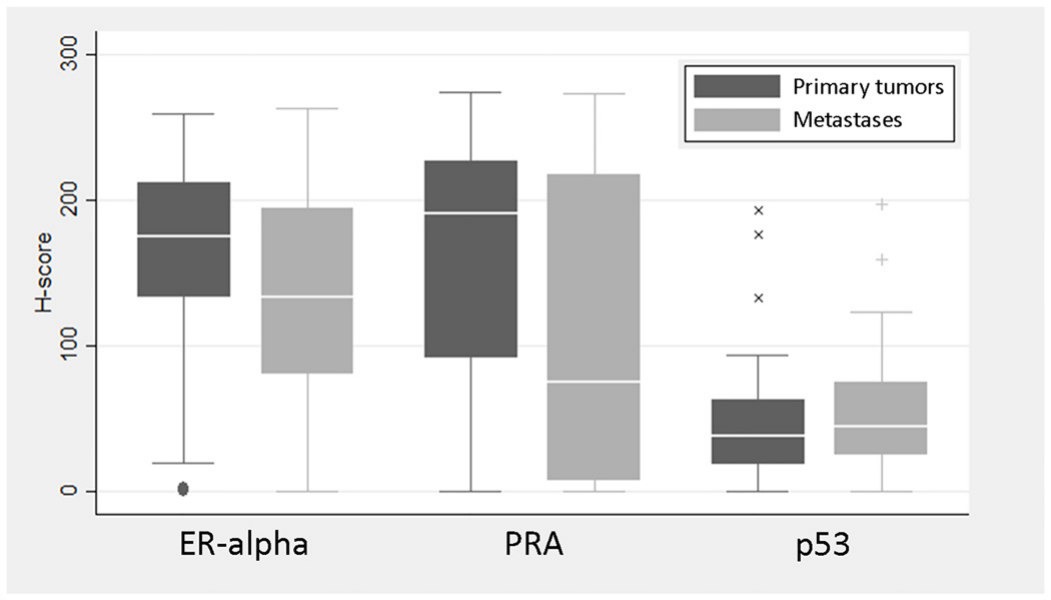
Immunohistochemical expression. Box-plots showing ER-alpha, PRA and p53 immunohistochemical expression levels in EEC primary tumors compared to metastases.

The morphological features of ECC primary tumors and metastases were similar in most of the cases (n = 31, 68.9%), but there were cases with discordant FIGO grade (n = 14, 31.1%) (Figs 1 and 2). The distribution of histological FIGO grade was significantly different when comparing metastases to matched primary tumors (p = 0.005). As described in Table 2 and Fig 3, metastases disclosed more frequently higher histological FIGO grade than primary tumors. On the other hand, mitotic rate was significantly lower in metastases when compared to matched primary tumors, with a median difference of -8 mitoses/10 HPF (p<0.001). This difference remains significant even when limiting the analysis to metastases at diagnosis (-8 mitoses/10 HPF, p<0.001).

**Table 2:** Comparison of morphological features and ER-alpha, PRA and p53 immunohistochemical expression between EEC primary tumors and metastases.

	Primary tumors	Metastases	Differences of matched pairs^a^	p
**Histological FIGO grade; n (%)**			14 (31.1%)	0.005
- Grade 1	16 (35.6%)	6 (13.3%)		
- Grade 2	12 (26.7%)	19 (42.2%)		
- Grade 3	17 (37.8%)	20 (44.4%)		
**Mitotic index (per 10HPF)**				<0.001
- median	32	23	-8	
- range	(4,153)	(0–114)	(-54, 20)	
**ER-alpha expression (H-score)**				0.002
- median	175.1	133.6	-21.9	
- range	(0.3, 259.2)	(0, 263.3)	(-238.4, 87.3)	
**PRA expression (H-score)**				<0.001
- median	191.2	75.1	-32.6	
- range	(0, 273.8)	(0, 273)	(-201, 75.1)	
**p53 expression (H-score)**				0.056
- median	38.4	44.5	2.5	
- range	(0, 244.8)	(0.1, 284)	(-47.3, 190.3)	

^a^ Differences were analyzed as number (%) of discordant cases for histological FIGO grade; and pairwise differences = metastasis value - primary tumor value for mitotic index and ER, PR, p53 H-score.

### Immunohistochemical ER-alpha, PRA and p53 expression

ER expression was intermediate to strong and diffuse in most EEC primary tumors, with a median staining extent of 77.1% positive cells and a median H-score of 175.1 (range: 0.3, 259.2). PR expression was also strong and diffuse in most EEC primary tumors, with a median staining extent of 71.5% positive cells, and a median H-score of 191.2 (range: 0, 273.8). There was a tendency for lower ER-alpha/PRA H-scores in EECs with higher histological FIGO grade. This was statistically significant for PRA (median PRA H-score by grade: I- 207.4, II-199.9, III-109.6; p = 0.022), but not significant for ER-alpha expression (median ER-alpha H-score by grade: I- 186.6, II-175.9, III-1355; p = 0.190).

The expression of p53 was within the levels considered as physiologic in the majority of EEC primary tumors, with scattered nuclei staining (median 19.9% positive cells, range: 0, 88.4%). The median p53 H-score was 38.4, ranging from 0 to 244.8. There was a tendency for higher p53 H-scores in EEC with higher histological FIGO grade, but not statistically significant (median p53 H-score by grade: I- 35.4, II-40.4, III-43.5; p = 0.343).

**Fig 4:**
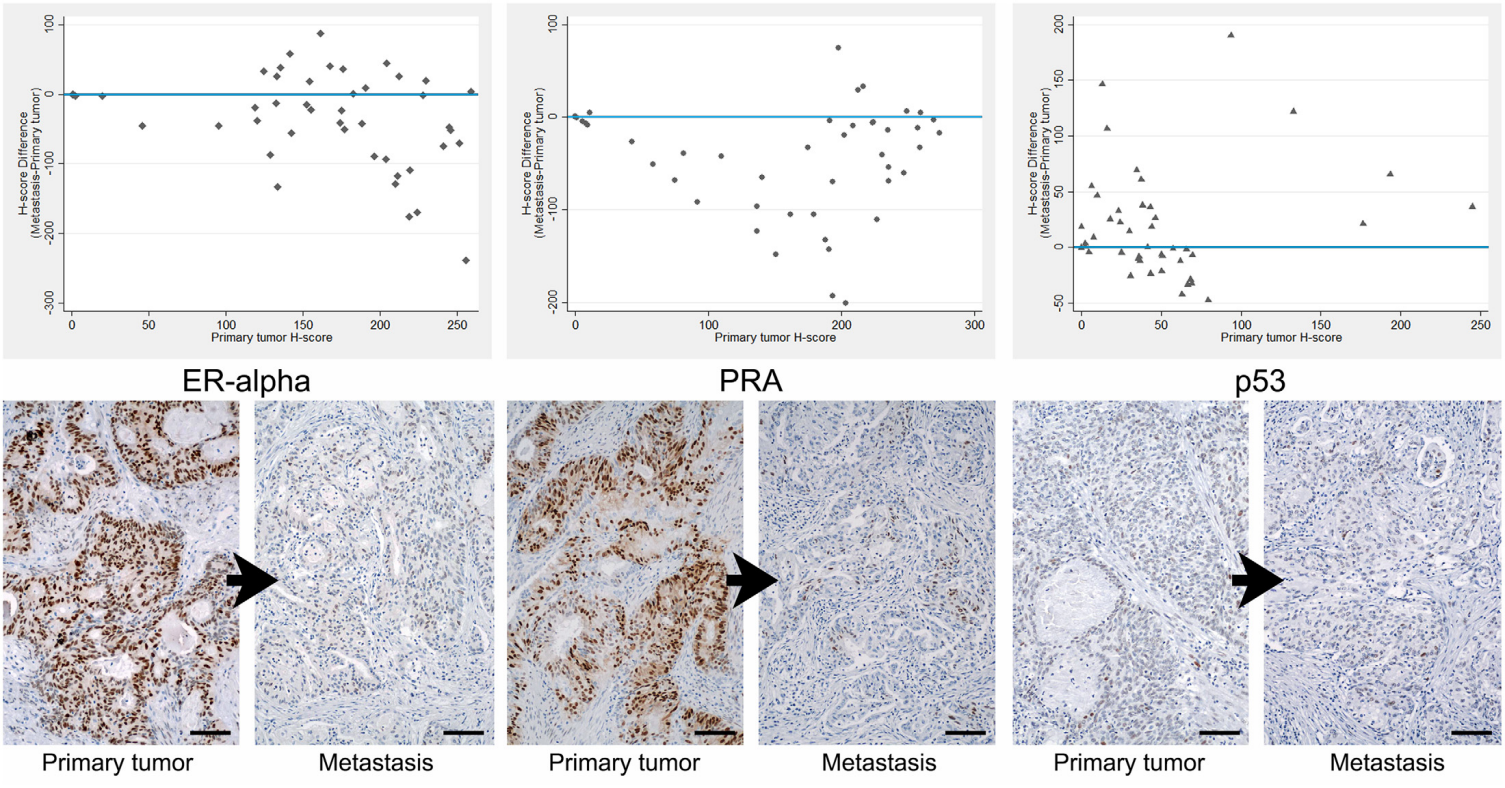
Immunohistochemical features. Scatter plots showing H-score differences in ER-alpha/PRA/p53 expression in EEC metastases matched to primary tumors and corresponding immunohistochemical representative examples. Plot horizontal bars corresponds to zero differences between primary tumors and metastases. It is evident that most cases showed a negative change of imunoexpression H-score, i.e. a decrease, of ER-alpha and PRA in metastases. Scale bar = 100μm.

The differences of expression between metastasis and primary tumor of each case are plotted in Fig 4. ER-alpha expression was significantly lower in metastases than in matched EEC primary tumors, with a median difference of -21.9 (p = 0.002). The same was observed when comparing PRA expression with a median difference of -32.6 (p<0.001). In contrast, for p53 there was no significant difference in expression between metastases and matched primary tumors (median difference = 2.5, p = 0.056). Additionally, the decreased expression in ER-alpha and PRA were correlated with each other (r_s_ = 0.58, p<0.001).

There was no relationship between the differences in ER-alpha and PRA expression and the differences found in tumor grade (ER-alpha: p = 0.783, PRA: 0.564) nor with differences found in mitotic index (ER-alpha: p = 0.906, PRA: p = 0.776). The PRA expression difference between metastases and primary tumors is significantly higher for FIGO stage I/II tumors compared to FIGO stage III/IV, but there was no association of ER-alpha and p53 expression with FIGO stage (ER-alpha: 0.079, PRA: 0.031, p53: 0.824). The decrease in ER-alpha and PRA expression also did not associate with the remaining clinicopathological parameters, namely metastasis site (ER-alpha: p = 0.397, PRA: p = 0.800), lymphovascular invasion (ER-alpha: p = 0.190, PRA: p = 0.764), radiotherapy (ER-alpha: p = 0.174, PRA: p = 0.211), chemotherapy (ER-alpha: p = 0.485, PRA: p = 0.883).

### ER-alpha and PRA gene promoter methylation levels

**Fig 5:**
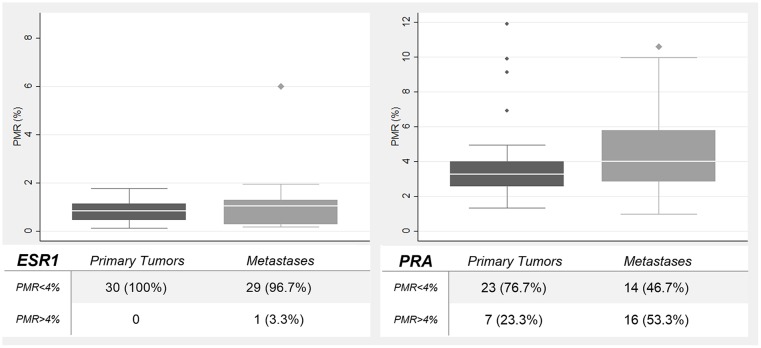
Gene promoter methylation levels. ER-alpha and PRA gene promoters methylation levels in EEC primary tumors compared to metastases.

Promotor methylation levels, expressed as PMR, of the genes studied (*ESR1*, *PRA*) were in general mild, varying from 0% to 11.9%. Using PMR as a continuous variable, there was a tendency for higher promoter methylation levels in metastases compared to primary tumors for *ESR1* (median PMR: primary tumors = 0.83% *vs*. metastases = 1.03%, p = 0.349), *PRA* (median PMR: primary tumors = 3.27% *vs*. metastases = 4.01%, p = 0.125), but without any statistical significance (Fig 5).

Analyzing dichotomized PMR, one (3%) of the metastases displayed *ESR1* gene promotor methylation, whereas no methylation of *ESR1* gene promotor was found in EEC primary tumors. Regarding PRA, there was a significant higher frequency of *its* promotor methylation in metastases (n = 16, 51.6%) compared to EEC primary tumors (n = 7, 22.7%) (p = 0.022).

There was no significant correlation between differences in immunohistochemistry expression and differences in methylation levels neither for estrogen nor progesterone receptors. However, the group of cases that showed a change in *PRA* promotor methylation status in metastases compared to the matched EEC primary tumors, displayed a larger difference in immunohistochemical PRA expression (median H-score difference = -41, range: -201, -4.6) compared to cases with no change in methylation status (median H-score difference = -13.8, range: -132.7, -75.1), but without statistical significance (p = 0.095). The metastasis with *ESR1* promoter hypermethylation did not express ER-alpha.

## Discussion

In our study we compared the morphology, ER-alpha, PRA and p53 immunoexpression of metastases and matched primary tumors in a series of 45 EECs. We showed that metastases had significantly higher FIGO grade, lower mitotic index and decreased ER-alpha and PRA expression compared to matched EEC primary tumors. No significant difference was found regarding p53 expression. Additionally, we found a higher frequency of hormone receptors gene promotor hypermethylation in metastases compared to matched primary tumors, particularly in *PRA* gene.

Very few studies have addressed the histological characterization of endometrial metastases/recurrences [17–19]. Soslow et al described a series of endometrial carcinomas in which discrepancies between recurrences and primary were found [18]. Particularly, they found a tendency for endometrial carcinomas to become morphological ambiguous or with higher grade in recurrences. Santacana et al. also commented on a higher histological grade in EEC vaginal recurrences of eight cases [17]. Our quantitative evaluation supports these findings showing a significant higher FIGO grade in 45 EEC metastases compared to matched primary tumors. This is in line with the hypothesis that metastases represent selected EEC primary tumor clones that acquire features associated with invasion and metastatic capability and/or tumor progression, as postulated by Darwinian tumor evolution models [24]. Noteworthy, histological FIGO grade may not always adequately indicate the metastatic potential of a heterogeneous tumor. Since FIGO grade relies on the proportional evaluation of aggressiveness-related features, namely solid pattern and cytological atypia, it is possible that low-grade FIGO tumors may harbor focal aggressive tumor clones.

Interestingly, in our study, metastases had a significant lower mitotic index than primary EEC. This finding might be due, in part, to the effect of cytotoxic agents used in EC adjuvant treatment, as reported in other cancer models [25, 26]. However, most (62.2%) of our cases comprised metastases detected at diagnosis, that displayed a significant decrease in mitotic index. In this setting it is possible that EEC metastatic tumor cells may have shifted their cellular mechanisms towards invasion/migration, as opposed to cell division, a concept often referred to as go-or-grow [27]. The proliferative activity of metastases remains controversial in cancer. While studies have reported a decrease in proliferation markers, e.g. breast and esophageal carcinoma lymph node metastases [28, 29], others suggested that metastases are more proliferative than matched primaries, e.g. omental ovarian cancer metastases [30]. In endometrial cancer, to the best of our knowledge, only one study evaluated Ki-67 in local vaginal recurrences and matched primaries, showing a significant higher Ki-67 expression in recurrences [17]. Importantly, a low mitotic index does not always imply a low proliferative activity, and mitotic index does not always correlates with Ki-67 expression [31].

ER and PR expression observed in our EEC primary tumors was similar to what has been reported in the literature, showing lower expression with higher tumor grade [32]. There was a significant decrease both in ER-alpha and PRA expression in metastases compared to primary tumors. Interestingly, there was no significant association between the decrease in hormone receptors and morphological changes. Soslow et al. also reported a frequent decrease of PR expression in matched primary and recurrent endometrial carcinomas; and in addition noted that there was no discordant morphology in the three EEC cases described as having PR expression discordance [18]. Though not statistically significant, Santacana et al. reported decreased expression of PR in recurrences compared to primary tumors [17]. Similarly, using unmatched endometrial carcinoma primary tumors and metastases Tangen et al. also reported a highest proportion of PR loss in the metastases [19]. The antibodies used in these three studies detected both PR isoforms. Fujimoto et al., using southern blot, described 3 cases of metastatic endometrial carcinoma in which PRA mRNA was suppressed compared to the primary tumor [33]. The same authors, in a later study showed that the ratio of ER-beta/ER-alpha immunohistochemical expression was increased in endometrial cancer metastases compared to primary tumors [34]. Interestingly, in breast carcinomas the discordance of hormone receptor status between metastases and primary tumors is fairly well documented [35–37]. Hormone receptor conversion by immunohistochemistry in distant breast cancer metastases occurs in a significant proportion patients, and has been shown to be more frequent for PR. Additionally, following the same trend as in breast cancer, hormone receptor expression changes in endometrial carcinoma may also have important implications for therapeutic strategies. EEC patients benefit from hormonal treatment, in particular those for which chemotherapy is not an option due to older age and comorbidities [5]. Nevertheless, the response rate to hormonal treatment reported in the literature is highly variable (11–56%), and usually poor [5]. This variation might be explained by the decrease of hormonal receptors in metastases as observed in the present study. Thus, assessment of hormone receptor status in recurrent/metastatic disease might prove more useful to tailor the treatment of patients with advanced EEC.

Regarding p53 expression we did not observe any significant differences between metastases and matched EEC primary tumors. While Soslow et al. also reported that p53 immunoexpression is usually concordant when evaluating matched pairs, with few exceptions, Santacana et al. reported that p53 expression was higher in patients with post-radiation recurrences [17, 18]. Thus, further studies are needed to clarify the role of p53 in EEC recurrences/metastases.

ER and PR changes during endometrial cancer progression most probably result from genetic, epigenetic or posttranslational alterations. In our study we searched for gene promoter DNA methylation as this is a well-recognized mechanism of gene silencing [38]. Globally, we found low ER and PR methylation levels in EEC primary tumors. However, when comparing primary tumors with metastases, using dichotomized PMR, statistically significant higher methylation levels were found for *PRA* in matched EEC metastases. This result is in agreement with the PR isoform A decrease protein expression observed in immunohistochemistry. There are only a few studies on ER and PR gene methylation in endometrial carcinomas, mainly using cell lines and rarely primary tumor samples. Reduced ER expression has not been consistently reported to be associated with ER genes promoter methylation [8, 9, 39]. Regarding PR, the functional role of its isoforms in the endometrium is not fully understood; nevertheless, some studies in knockout mice reported that PRA expression is necessary to mediate the anti-proliferative responses to progesterone and to inhibit estrogen-induced proliferation [13]. Additionally, PRA has been also shown to function as a transcriptional inhibitor of other steroid hormone receptors, such as ER and PR-B [40]. Hence, it is possible that a decrease in PRA isoform expression and its gene promoter methylation might play a role in tumor progression. *PRA* methylation was associated with progestin-resistance in breast carcinoma [41]. *In vitro* studies have suggested that in endometrial carcinoma it is the PRB isoform that plays a major role in carcinogenesis, by regulating anti-proliferative activity and invasiveness [42, 43]. PRB isoform, compared to PRA, seems to be underexpressed in high-grade endometrial carcinoma, but loss of both PR isoforms has been associated with poor prognosis [14, 15, 44, 45]. Noteworthy, other mechanisms were also shown to alter PR function in endometrial carcinoma, such as complex of nucleic acid sequence aberrations (e.g. PROGINS) [46], *PR* promoter polymorphisms [46, 47], and exon region methylation [12, 48]. Moreover, there is recent evidence that PRA also plays a major role in endometrial carcinogenesis, namely by decreasing PR transcription and respective expression in the stroma of progesterone refractory endometrial carcinomas [49].

Some limitations of our study include possible selection bias since we only analyzed EEC metastases that have been biopsied or excised. Furthermore, the evaluation of ER/PR immunohistochemical expression might have been affected by intratumoral heterogeneity; however, most EEC usually showed homogeneous staining. Also, we should mention that the antibodies used were specific only for ER alpha and PRA isoform. Even though studying PRB and ER-beta expression would allow a broader knowledge of hormonal status in EEC metastatic progression, our results clearly showed that ER-alpha and PRA are deregulated in that process.

The apparent discrepancies regarding methylation results obtained by us and those reported by others might be due to the different sensitivities of the method/primers used and to the stringent criteria in the analysis.

## Conclusion

EEC metastatic progression seems to be related to loss of ER-alpha and PRA expression, but does not appear to depend on the increase of p53 expression. Conventional morphological markers of EEC aggressiveness such as histological FIGO grade are relevant in EEC metastases, but do not associate with ER-alpha and PRA loss in metastases. The decrease in PRA expression highlights that, for patients candidates to hormone therapy, receptor status assessment might be more informative in metastases than in EEC primary tumors. Gene promotors’ aberrant methylation seems to play a limited role in the decrease of PRA expression in metastatic EEC.
